# Fostering responsible research with genome editing technologies: a European perspective

**DOI:** 10.1007/s11248-017-0028-z

**Published:** 2017-07-20

**Authors:** Hervé Chneiweiss, François Hirsch, Lluis Montoliu, Albrecht M. Müller, Solveig Fenet, Marion Abecassis, Jennifer Merchant, Bernard Baertschi, Mylène Botbol-Baum, James A. Houghton, Mihalis Kritikos, Janet Mifsud, Ewa Bartnik, Johannes Rath, Christiane Druml, Bärbel Friedrich, Ana Sofia Carvalho, Dirk Lanzerath, Agnès Saint-Raymond

**Affiliations:** 1 INSERM Ethics Committee, Paris, France; 2 National Centre for Biotechnology (CNB-CSIC), CSIC Ethics Committee and CIBERER-ISCIII, Madrid, Spain; 3 Institute of Medical Radiology and Cell Research, University of Würzburg, Wūrzburg, Germany; 4 University Panthéon-Assas, Paris, France; 5 University of Geneva, Geneva, Switzerland; 6 Catholic University of Louvain, Louvain, Belgium; 7 National University of Ireland, Galway, Ireland; 8 Research Group on Law, Science, Technology and Society (LSTS), Vrije Universiteit Brussel, Ixelles, Belgium; 9 University of Malta, Msida, Malta; 10 Faculty of Biology, Institute of Genetics and Biotechnology, University of Warsaw, Warsaw, Poland; 11 University of Vienna, Vienna, Austria; 12 UNESCO Chair on Bioethics, Medical University of Vienna, Vienna, Austria; 13 German National Academy of Sciences Leopoldina, Halle, Germany; 14 Instituto de Bioética, and Portuguese National Council for Ethics in Life Sciences, Universidade Católica Portuguesa, Porto, Portugal; 15 European Research Ethics Committee Network (EUREC), Ixelles, Belgium; 16 Head of Human Medicines Special Areas, European Medicines Agency, London, UK

**Keywords:** CRISPR-Cas, Gene editing, Science and society, Responsible research and innovation

## Abstract

In this consensus paper resulting from a meeting that involved representatives from more than 20 European partners, we recommend the foundation of an expert group (European Steering Committee) to assess the potential benefits and draw-backs of genome editing (off-targets, mosaicisms, etc.), and to design risk matrices and scenarios for a responsible use of this promising technology. In addition, this European steering committee will contribute in promoting an open debate on societal aspects prior to a translation into national and international legislation.

For several years, scientists have been trying to develop techniques to specifically target and modify sequences within complex genomes. New technologies that allow the specific addition, removal, or modification of DNA sequences are summarized under the term ‘genome editing’ (Gaj et al. 2013). If the genome edited sequence corresponds to a gene, then the amino-acid sequence of the protein encoded by the gene may be altered. In some cases, this may lead to changes in its activity and function, as well as its location or lifespan. Thereby, genome editing may result in the correction of a defective function of a gene within a specific biological context. The latest advance in genome editing by CRISPR (clustered regularly interspaced short palindromic repeats)/Cas (Mojica and Montoliu 2016), is unquestionably a major technological revolution. This is illustrated by the rapid expansion of the scientific literature on CRISPR/Cas9-mediated genome editing. More than 3000 peer-reviewed articles citing “CRISPR or Cas9” had been published by January 2017 (https://www.ncbi.nlm.nih.gov/pmc/articles/PMC5064173/). There is also a continuing emergence of novel related tools which are potentially more efficient than CRISPR-Cas9 (Barrangou and Doudna 2016) such as Cas12a (Cpf1) (Zetsche et al. 2015). The economic potential of gene editing seems enormous and major companies are investing millions of euros in CRISPR-Cas9. In parallel, large numbers of patents have been filed and there are ongoing disputes over patents and licensing rights (http://www.nature.com/news/titanic-clash-over-crispr-patents-turns-ugly-1.20631), the outcomes of which could be worth billions of euros.

CRISPR-Cas9 is a genome editing tool that is able to induce a double-strand break into DNA at selected sites in the genome of any cell and specy. In practice, a guide RNA (gRNA) leads the DNA endonuclease Cas9 to a specific sequence to instruct a cut through the DNA strands (Braff et al. 2016). The gRNA must be homologous (complementary) to the desired target sequence and then Cas9 binds to the chosen genomic locus close to a short DNA sequence motif called PAM (protospacer adjacent motif). The Cas9 enzyme cuts through the DNA creating a double-strand break. The cell may then use different mechanisms to repair the break. These include DNA repair systems present in all cells and result in non-homologous end joining (NHEJ), or by homology-directed repair (HDR). As a result, sequence modifications are introduced at the break site (insertion, deletion or mutation). If the objective is to knock-down the expression of the targeted gene, it is sufficient to allow the NHEJ repair system to mend the break by inserting and/or deleting (INDELs) nucleotides randomly. As the repair is error-prone, the “repaired gene” will most likely be mutated. If the objective is to correct a pre-existing mutation, then the repair must restore the original sequence after the break of the mutated gene. For this to happen, the introduction of a template DNA sequence is necessary and the cell repairs the break by copying the template sequence. The same applies to introducing a mutation that mimics a variant of a gene. It is also possible to simultaneously modify multiple targets. Of note, the repairing mechanisms will usually trigger the generation of multiple and diverse edited alleles, and hence normally lead to mosaicism in cells or animals. Interestingly, it was recently shown that a bacteriophage protein can switch-off the CRISPR/Cas9 activity, which should permit a certain level of control of CRISPR/Cas9-mediated gene editing, although this approach does not revert a modification already initiated (Rauch et al. 2017).

The simplicity of carrying out this procedure enabled the pioneers of genome editing technology, such as George Church from Harvard, to declare that the technique could “on a simple whim allow anyone to do almost everything”. Furthermore, the Church team described orthologs of Cas9 with improved selectivity, specificity and efficiency of targeting a particular DNA sequence (Braff et al. 2016). Beyond coding and non-coding DNA, targeted modifications of the epigenome at specific sites, particularly for therapeutic purposes, are now feasible.

Almost all areas of biological research are, or will soon be penetrated by the rapid emergence and development of genome editing technologies. With respect to humans, genetic changes of somatic cells, germ cells or embryos are clear targets for these new approaches. However, most of the therapeutic strategies are expected to be developed for somatic (or ex vivo) gene-therapy approaches, not involving embryos (http://www.nature.com/news/crispr-gene-editing-tested-in-a-person-for-the-first-time-1.20988). As regards non-human animals, both livestock and laboratory animals are candidates for these new methodological approaches. Environment and biodiversity are also clearly among the potentially affected areas. Gene drive approaches (Gantz and Bier 2015) could be applied for pest control where a CRISPR-Cas9 cassette is able to self-perpetuate, thereby rapidly spreading any genetic information among all individuals of a population. This possibility also raises concerns about potential misuse and that gene editing technologies may be used for the development of genetic weapons of mass destruction (https://www.dni.gov/files/documents/SASC_Unclassified_2016_ATA_SFR_FINAL.pdf).

Therefore, together, these new possibilities lead us to consider the use of CRISPR-Cas9 technology in the light of the regulation that currently frames and oversees contemporary genomic technologies, and how they might incline us to reconsider these regulations. The same questions are raised by related genome editing tools with similar possibilities, including engineered meganucleases, zinc finger nucleases (ZFN) and transcription activator-like effector nucleases (TALEN).

Several academic institutions such as the US NAS/NAM (http://nationalacademies.org/gene-editing/consensus-study/meetings/index.htm#slides3) and, more recently, the European Academies Science Advisory Council (EASAC) (https://www.knaw.nl/shared/resources/internationaal/bestanden/easac-report-31) addressed the ethical, legal and social aspects (ELSA) raised by these new genome editing tools. Based on its report published December 2015, the INSERM Ethics Committee organised a meeting in Paris on March 16th, 2016, with a wide range of European stakeholders and experts to reflect on, and foster, responsible research with CRISPR-Cas (http://www.inserm.fr/inserm/accueil/qu-est-ce-que-l-inserm/l-ethique-a-l-inserm/seminaires-du-comite-d-ethique/atelier-du-comite-d-ethique-inserm-fostering-responsible-research-with-crispr-cas9/(language)/eng-GB). Consensus recommendations are captured in the following position outlined below. Obviously, due to the rapid scientific advances in this field, these principles will most likely require further modification in the future.

As the situation currently stands, no international consensus exists, similar to the one that resulted from the ‘Asilomar Conference on Recombinant DNA’ in 1975, although a recent proposal did debate the possibility of calling for an international ban on the gene-drive approach (Callaway 2016). We consider that a moratorium is not appropriate to promote good basic research practice and adequate safeguards. Of note, the new genome editing techniques do not raise fundamental new biological risks that have not already been encountered by existing technologies. However, since performing genome editing by the new tools is much easier, cheaper and faster than with the previously available technologies; these new applications must be thoroughly assessed.

Since basic research in the area should be permitted to continue, we propose that the following general principles should be adopted:

1. *To foster research that will assess the feasibility, the efficacy and the safety of genome editing techniques*, such as the benefit-to-harm balance of any potential clinical application can be evaluated. It is necessary to evaluate the efficacy of genome editing techniques, to estimate the impact of mosaicism at the on-target location, potential off-targets and of other adverse effects and to assess their clinical relevance. This task is essential in order to define what therapeutic approaches should be considered for use in humans, and which research institutions would then promote for these studies to be conducted according to standardized methods.

This aim could be addressed by establishing a European Steering Committee (ESC) gathering experts from a broad spectrum of relevant disciplines as diverse as molecular and cell biology, ecology, safety and a variety of social sciences, to evaluate:

- Acceptable levels and types of off-target effects,
- Acceptable levels of mosaicism,
- Acceptable levels of epigenetic effects.

The ESC should rely on an open and transparent discussion process which should include various stakeholders, for example patient organizations, representatives of Ethics committees and of the economic sector, as well as representatives of the communication sector.

2. *To evaluate the potential adverse effects of gene drive applications* with a thorough risk assessment analysis and mitigated before environmental trials are undertaken outside the laboratory. These field exercises should be conducted using strict confinement precautions similar to those that have already been developed for infectious and GMO´s approaches. Given the transmissible nature of gene drive genetic elements, as well as the irreversibility of genetic errors that may occur, assessments will have to be made over a long time period. Research on plausible risks should be developed. Measures will have to be foreseen in the event of unexpected adverse effects.

With a well thought-out procedure for the assessment of a benefit-to-harm balance in the long-term, the proposed European Steering Committee will produce risk analysis matrices, devise realistic scenarios and will produce recommendations for reversibility strategies in the case of adverse effects harmful for humans or for biodiversity.

3. *To reassess the ban on all modifications of the germ line nuclear genome for clinical application in human reproduction* Many European countries have ratified the Oviedo Convention of the Council of Europe (http://www.coe.int/en/web/conventions/full-list/-/conventions/treaty/164), including its article 13 that is relevant to germ line genome editing. An open discussion is needed on a case-by-case analysis for a restricted number of genetic disorders, such as Huntington’s disease that may be prevented by genome editing, as well as other very rare diseases for which we have no therapy. At the present time, there must be opposition to any demands for the modification of the related legal framework, in so far as clinical applications are concerned, until uncertainty about potential harms has been evaluated on the basis of research, and until consensus has been reached with multiple partners throughout civil society. Again, it is important that society maintains a broad confidence in science. This requires an appropriate oversight of laboratory work and of any medical and ecological application of genome editing techniques especially if it is irreversible and permanent.

European research institutions and political decision-makers should cooperate in the definition of ethical standards and guidelines which determine what kinds of translational research and application of genome editing are admissible and are not.

4. *To be pro-active to prevent this technology from being hijacked by those with extremist views and to avoid misleading public expectation with overinflated promises* Unlike many other new technologies applied to genetics, the new genome editing approaches indeed offer almost unlimited possibilities. Therefore, the scientific community must act with responsible openness and transparency. A major issue is to distinguish between the questions and concerns raised by the application of genome editing technologies in research, and their clinical application in patients. The role of legal measures is of considerable importance in this discussion in order to build a consensus given the high scientific uncertainty, the potential misuses and security risks, the ethical tensions, the conflicting interests and the rapid developments in this scientific area.

European research institutions should contribute to national and international initiatives addressing questions of freedom of research and of medical ethics. Participation in such international initiatives by experts from developing countries should be promoted and facilitated, since all countries worldwide are concerned and potentially be affected. International biorisk management as an inclusive approach to safety and security should be expanded to cover the unique risks related to safety and security in the context of genome editing.

5. *To raise awareness about the distinction between the care/treatment of human diseases and human enhancement* Certain therapeutic promises might engender dystopian expectations. As such, animated discussion about controversial technological advances in the life sciences is a very effective means of heightening public interest in research and embeds science at the heart of public culture. We must indeed foster increased debate within the scientific community and with the rest of civil society aiming at contributing to the advancement of a necessary global responsible scientific research and innovation.
